# Dicke and Fano-Andreev reflections in a triple quantum-dot system

**DOI:** 10.1038/s41598-021-83407-6

**Published:** 2021-02-16

**Authors:** A. González I., M. Pacheco, A. M. Calle, E. C. Siqueira, P. A. Orellana

**Affiliations:** 1 Departamento de Física, Universidad Técnica Federico Santa María, 110 V Valparaíso, Casilla Chile; 2 Departamento Acadêmico de Física, Universidade Tecnológica Federal do Paraná, Curitiba, Brazil

**Keywords:** Atomic and molecular physics, Condensed-matter physics, Quantum physics, Mathematics and computing, Nanoscience and technology, Physics

## Abstract

This article studies quantum interference effects and their influence on the electronic transport through a parallel triple quantum-dot system coupled to normal and superconducting leads in the linear response and non-equilibrium regime. We model the system by a triple impurity Anderson Hamiltonian including the Coulomb intra-dot correlations in all quantum-dots. Using the non-equilibrium Green’s function formalism, we calculate the Andreev conductance and the transmittance for energies within the superconductor gap. Our results show that the Andreev reflection spectra, both in the presence and absence of Coulomb interaction, reveal Fano and Dicke-like resonances in analogy to the Fano and Dicke effects in atomic physics. As one of the main results, we obtain that the charge shows abrupt changes due to the Dicke effect.

## Introduction

Electron transport through multiple quantum dots systems exhibits exciting interference effects such as Fano^1–7^ and Aharonov-Bohm^8–12^. The interference phenomenon, which resembles the well-known Dicke resonance in atomic physics, appears to be of particular importance^13^. It manifests itself by a narrow (subradiant) and a broad (superradiant) line-shape, spontaneously emitted by closely linked atoms, separated by a distance smaller than the wavelength of the emitted light^14^. In the electronic case, the decay rates (level broadening) are produced by the couplings between localized levels and the conduction channel, and the proximity and effective couplings give rise to fast (super-tunneling) and slow (sub-tunneling) modes^15^.

The presence of the Dicke effect has been predicted theoretically and experimentally in different nanoscopic systems^16–24^. On the other hand, Fano effect is another quantum interference phenomena that has been studied in quantum transport for some time. This effect is due to the quantum interference from localized states and continuum states. It yields characteristic asymmetric Fano lineshape, characterized by the Fano factor *q*, which is a measure of the coupling strength between the continuum and the localized state.

The electronic transport through quantum dots (QDs), double quantum dots (DQDs) and triple quantum dots (TQDs) coupled to normal/ferromagnetic and superconductor leads, has been studied recently^25–30^. The Cooper pairs^31^ transport, along with interference effects among electrons and holes, gives rise to novel and interesting phenomena^32,33^. Within this context, several features of the Dicke effect have been considered under the presence of superconductor correlations. In particular, it has been found that the Dicke effect occurs in the Andreev conductance spectrum by modulating the interdot coupling and the side-dot levels^34,35^. A description of the relationship between the induced electron pairing and the Dicke effect has been studied^36^ by focusing specifically on how the electron pairing and correlation effects are affected by the side-attached quantum dots, ranging from the interferometric to the molecular limits. However, a detailed description of the interplay between Dicke effects and the charging effect induced by the Coulomb intra-dot correlations, is still missing.

In this paper, we present an investigation of the influence of the Dicke and Fano effects on electronic transport through a coupled triple quantum dot system coupled to normal and superconducting leads in a linear and non-linear regime.

In particular, we study the interplay between the proximity effect due to the superconducting lead and the conjunction of two phenomena: Dicke and Fano-Andreev reflections. We shall focus on the electronic properties of TQDs within the regime of low temperatures and sub-gap energies $(eV\ll \Delta )$, where the electronic transport is mainly carried by Andreev reflection (AR). We consider Coulomb correlations in all the quantum-dots and study their influence on the electronic conductance. The inter-dot Coulomb interaction is assumed to be much smaller than the corresponding intra-dot interaction and, in consequence, is omitted. Transport characteristics, such as the Andreev conductance and the electrical current of the system at the low-temperature limit, are derived using the non-equilibrium Green function method, in the linear and non-linear response regime. We use the Hubbard I approximation^37^ to obtain the relevant Green’s functions from the equations of motion. Our results show that the Andreev reflection spectra, both in the presence and absence of Coulomb interaction, reveal Dicke and Fano-like resonances in analogy to their counterpart effects in atomic physics. As one of the main effects, we obtain that the charge shows abrupt changes due to the Dicke effect, which has not been shown in previous reports.

The paper is organized as follows. “Results” introduces the model and describes the general background of the transport properties in the TQD system. Next, “Summary” presents the corresponding numerical results in the equilibrium and non-equilibrium regime for both non-interacting and interacting cases. Finally, “Appendix” closes with a summary and general conclusions.

## Results

**Figure 1:**
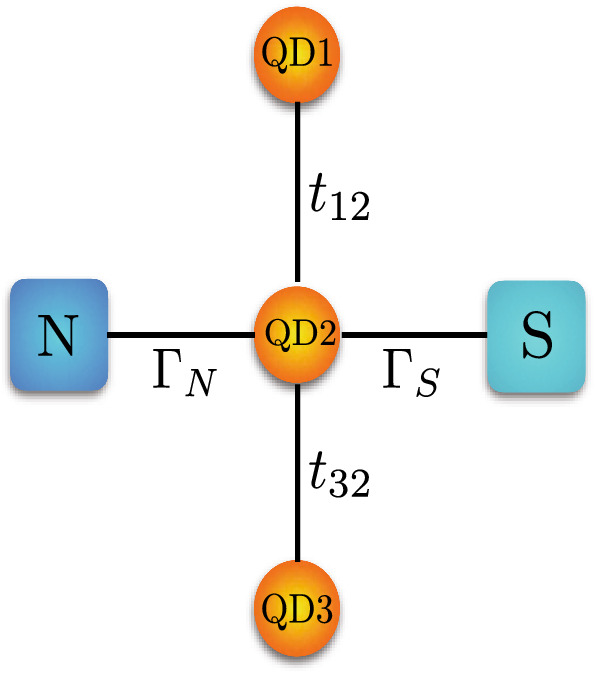
A sketch of triple-QD system coupled to normal metal and superconducting leads.

## Model

The system under consideration consists of a single-level central quantum dot (QD2) attached to one normal metallic and one superconducting lead and two side quantum dots (QD1 and QD3) as shown schematically in Fig. 1. The following Hamiltonian models the system (N-TQD-S):

1 $$\begin{aligned} H = H_{N} + H_{S} + H_{TQD} + H_{T}. \end{aligned}$$

The first term is the Hamiltonian for the normal electrode and it is given by:

2 $$\begin{aligned} H_{N} = \sum _{k} \sum _{\sigma } \epsilon _{k N \sigma } c^{\dagger }_{k N \sigma } c_{k N \sigma }, \end{aligned}$$

where $c_{k N \sigma }^{\dag }$ ($c_{k N \sigma }$) is the electron creation (annihilation) operator of an electron with spin $\sigma$ and energy $\epsilon _{kN\sigma }$ in the normal electrode.

The second term stands for the *BCS* Hamiltonian^31^ of the superconducting (right) lead and reads:

3 $$\begin{aligned} H_{S} = \sum _{k} \sum _{\sigma } \epsilon _{kS} c^{\dagger }_{k S \sigma } c_{k S \sigma } + \sum _{k} (\Delta ^{*} c_{k S \downarrow }c_{-k S \uparrow } + \Delta c^{\dagger }_{-k S \uparrow } c^{\dagger }_{k S \downarrow }), \end{aligned}$$

where $c_{k S \sigma }^{\dag }$ ($c_{k S \sigma }$) is the electron creation (annihilation) operator of an electron with spin $\sigma$ and energy $\epsilon _{kS}$ in the superconducting electrode, and $\Delta$ denotes the superconducting energy gap.

The third term is the Hamiltonian of three coupled *QDs*, given by

4 $$\begin{aligned} H_{TQD} = \sum _{m, \sigma } \epsilon _{d m, \sigma } d^{\dagger }_{m, \sigma } d_{m, \sigma } + \sum _{\sigma } \sum _{j=1,3} \left[ t_{j2} d^{\dagger }_{2 \sigma } d_{j \sigma } + H.c \right] + \sum _{m} U_m n_{m \sigma } n_{m \bar{\sigma }}, \end{aligned}$$

where $d_{m \sigma }^{\dag }$ ($d_{m \sigma }$) is the electron creation (annihilation) operator of an electron with spin $\sigma$ and energy $\epsilon _{d m}$ in the *m*-th quantum dot with $m = 1,2,3$. We assume that each quantum dot has only a single-electron spin-degenerate level, with $\epsilon _{d m \uparrow } = \epsilon _{d m \downarrow }$; $U_m$ is the strength of the Coulomb interaction in the *m*-th quantum dot while $t_{j2} (j = 1,3)$ stands for the interdot coupling parameter.

The last term in Eq. (1) describes tunneling of electrons between the leads (*N*, *S*) and the central quantum dot (*QD*2) :

5 $$\begin{aligned} H_T= \sum _{k \sigma } \sum _{\beta =N, S} (V_{k\beta \sigma } c^{\dagger }_{k \beta \sigma } d_{2 \sigma } + H.c), \end{aligned}$$

where $V_{k\beta \sigma } (\beta = N, S)$ is the tunneling matrix element between the central *QD*2 and the electrode $\beta$. In the wide-band limit approximation, one can introduce the coupling constants with the leads: $\Gamma _{\beta } = 2 \pi \sum _k |V_{k\beta }|^2 \delta (\omega - \epsilon ^{\beta }_{k})$.

The retarded Green’s function $\mathbf {G}^{r}_{\sigma }$ in the generalized $6 \times 6$ Nambu representation is obtained from the Dyson equation:

6 $$\begin{aligned} \mathbf{G}^{r} = \mathbf{g}^r + \mathbf{g}^r {\varvec{\Sigma }}^r \mathbf{G}^{r}, \end{aligned}$$

where $\mathbf{g}^{r}$ denotes the retarded Green’s function of the triple quantum dot isolated from the leads being written as:

7 $$\begin{aligned} \mathbf{g}^{r} = \begin{bmatrix} \left[ \mathbf{g}_{ 1}^{r} \right] ^{-1} &{} \mathbf{t} &{} \mathbf{0} \\ \mathbf{t^{*}} &{} \left[ \mathbf{g}_{ 2}^{r} \right] ^{-1} &{} \mathbf{t} \\ \mathbf{0} &{} \mathbf{t^{*}} &{} \left[ \mathbf{g}_{ 3}^{r} \right] ^{-1} \end{bmatrix}^{-1}, \end{aligned}$$

where $\mathbf{g}_{m}^{r}$ matrices are defined as:

8 $$\begin{aligned} \mathbf{g}^{r}_{ m} = \begin{bmatrix} \dfrac{1 - n_{m }}{\omega - \epsilon _{d m}} + \dfrac{ n_{m }}{\omega - \epsilon _{d m} - U_m} &{} 0 \\ 0 &{} \dfrac{1 - n_{m}}{\omega + \epsilon _{d m }} + \dfrac{ n_{m }}{\omega + \epsilon _{d m} + U_m} \end{bmatrix}, \end{aligned}$$

with $m = 1,2,3$. The interdot coupling matrix $\mathbf{t}$ is given by:

9 $$\begin{aligned} \mathbf{t}= \begin{bmatrix} -t &{} 0 \\ 0 &{} t \end{bmatrix}. \end{aligned}$$

The retarded self-energy of the leads, in the wide-band approximation, acquires the following form:

10 $$\begin{aligned} {\varvec{\Sigma }}^{r}= \begin{bmatrix} \mathbf {0} &{} \mathbf {0} &{} \mathbf {0} \\ \mathbf {0} &{} { \varvec{\Sigma }}^{r}_{N } + {\varvec{\Sigma }}^{r}_{S } &{} \mathbf {0} \\ \mathbf {0} &{} \mathbf {0} &{} \mathbf {0}, \end{bmatrix} \end{aligned}$$

where

11 $$\begin{aligned} {\varvec{\Sigma }}^{r}_{N} = -\frac{i}{2} \Gamma _N \begin{bmatrix} 1 &{} 0 \\ 0 &{} 1 \end{bmatrix} \end{aligned}$$

and

12 $$\begin{aligned} {\varvec{\Sigma }}^{r}_{S }= -\frac{i}{2} \rho _s (\omega ) \Gamma _S \begin{bmatrix} 1 &{} - \frac{\Delta }{\omega } \\ - \frac{\Delta }{\omega } &{} 1 \end{bmatrix}, \end{aligned}$$

where $\rho _s$ denotes the dimensionless modified BCS density of states in the superconductor given by:

13 $$\begin{aligned} \rho _{s}(\omega ) = \frac{| \omega | \theta (| \omega | - \Delta )}{\sqrt{ \omega ^2 - \Delta ^2}} - i \frac{\omega \theta (\Delta - | \omega | )}{\sqrt{ \Delta ^2 - \omega ^2 }}. \end{aligned}$$

We have adopted the equation of motion method and the Hubbard-I decoupling scheme to find the Green’s functions. The general expression for the charge current through a barrier from the normal lead to the *QDs* can be calculated in terms of non-equilibrium Green’s function $G^{r,a}$. The charge current *I* flowing in a biased system from left to right can be calculated from the following expression:

14 $$\begin{aligned} I = - e \left\langle \frac{d N}{dt} \right\rangle \end{aligned}$$

with $$N = \sum _{k \sigma } c^{\dagger }_{k \sigma } c_{k \sigma }$$.

By using the equation of motion (EOM), we can obtain

15 $$\begin{aligned} I = \frac{2 e}{\hbar } \sum _{} \int \, d\omega \, [ \mathbf{G}^{r}_{2}(\omega ) {\varvec{\Sigma }}^{<}_{N}(\omega ) + \mathbf{G}^{<}_{2}(\omega ) {\varvec{\Sigma }}^{a}_{N }(\omega ) + H.c. ]_{(11)}, \end{aligned}$$

where $\mathbf {G}^{r, <}_{}(\omega )$ is the Fourier transform of retarded and lesser Green’s function of the system, and $\varvec{\Sigma }^{<, a}_{N}$ is the Fourier transform of lesser, advanced self-energy of the normal lead.

In order to obtain the lesser Green’s function $G^{<}_{\sigma }(\omega )$, we use the Keldysh equation

16 $$\begin{aligned} \mathbf {G}^{<}_{} = \varvec{G}^{r}_{}(\omega ) \varvec{\Sigma }^{<}_{} \mathbf {G}^{a}_{}(\omega ), \end{aligned}$$

where the lesser self-energy is given by

17 $$\begin{aligned} {\varvec{\Sigma }}^{<}_{}(\omega ) = {\varvec{\Sigma }}^{<}_N + {\varvec{\Sigma }}^{<}_S = \begin{bmatrix} \mathbf {0} &{} \mathbf {0} &{} \mathbf {0} \\ \mathbf {0} &{} \mathbf{f}_N(\omega ) {\varvec{\Gamma }}^{}_{N} + \mathbf{f}_S(\omega ) {\varvec{\Gamma }}^{}_{S} &{} \mathbf {0} \\ \mathbf {0} &{} \mathbf {0} &{} \mathbf {0}, \end{bmatrix} \end{aligned}$$

while $\mathbf {f}_i$ is the Fermi matrix, given by

18 $$\begin{aligned} \mathbf{f}_i= \begin{bmatrix} f_i &{} 0 \\ 0 &{} \bar{f}_i \end{bmatrix} \end{aligned}$$

with the Fermi functions for electrons and holes defined as $f_i = f (\omega - V_i )$ and $\bar{f}_i = f (\omega + V_i )$, respectively. For $i = N$ and $f_i = \bar{ f}_i = f (\omega )$ for $i = S$. Also,

19 $$\begin{aligned} {\varvec{\Gamma }}^{}_{N}= \begin{bmatrix} \Gamma _N &{} 0 \\ 0 &{} \Gamma _N \end{bmatrix} \end{aligned}$$

and

20 $$\begin{aligned} {\varvec{\Gamma }}^{}_{S}= \bar{\rho }_s (\omega ) \Gamma _S \begin{bmatrix} 1 &{} - \frac{\Delta }{\omega } \\ - \frac{\Delta }{\omega } &{} 1 \\ \end{bmatrix} \end{aligned}$$

denotes the couplings constants in the matrix form of the leads, while $\bar{\rho }_{s}$ denotes the density of states in the superconductor given by

21 $$\begin{aligned} \bar{\rho }_s(\omega ) = \frac{| \omega | \theta (| \omega | - \Delta )}{\sqrt{ \omega ^2 - \Delta ^2}}. \end{aligned}$$

Finally, by substituting the matrix elements previously calculated, the current in the sub-gap regime ($e |V| < \Delta$), i.e., the Andreev current in the limit of low temperature, can be written as

22 $$\begin{aligned} I_{A}= \frac{ 2 e}{h} \int _{- e \, V}^{ e \, V} \, d\omega \, T_{A}(\omega ), \end{aligned}$$

where *V* is the bias voltage, and $T_A$ is the Andreev transmittance, given by:

23 $$\begin{aligned} T_A = \Gamma _N^2 |G^{r}_{2,12}(\omega )|^2. \end{aligned}$$

It is important to note that the Coulomb correlations make $T_{A}$ dependent on the average occupations of the QDs. For the non-magnetic case, the averaged occupation number does not depend on spin, which allows us to set for each QD: $\langle n_{i, \sigma } \rangle = \langle n_{i} \rangle$, $i = 1,2,3$. These occupation numbers are obtained by solving the following system of equations:

24a $$\begin{aligned} \langle n_{1} \rangle= & {} - i \int \frac{d \omega }{2 \pi } \, G^{<}_{1,11}[\omega , \langle n_{1}\rangle ,\langle n_{2}\rangle ,\langle n_{3}\rangle ], \end{aligned}$$

24b $$\begin{aligned} \langle n_{2} \rangle= & {} -i \int \frac{d \omega }{2 \pi } \, G^{<}_{2,11}[\omega , \langle n_{1}\rangle ,\langle n_{2}\rangle ,\langle n_{3}\rangle ], \end{aligned}$$

24c $$\begin{aligned} \langle n_{3} \rangle= & {} -i \int \frac{d \omega }{2 \pi } \, G^{<}_{3,11} [\omega , \langle n_{1}\rangle ,\langle n_{2}\rangle ,\langle n_{3}\rangle ]. \end{aligned}$$

As one may notice by inspecting Eqs. (24a), (24b) and (24c), they form a system of equations for $\langle n_{1}\rangle$, $\langle n_{2}\rangle$ and $\langle n_{3}\rangle$ which must be solved in a self-consistent way.

The LDOS of the quantum dots come from of matrix elements of the retarded Green’s function matrix (electron components in Nambu space). The LDOS for dots 1, 2 and 3 are, respectively:

25a $$\begin{aligned} \rho _{1}(\omega )= & {} - \frac{1}{ \pi } Im ( G^{r}_{1,11}[\omega , \langle n_{1}\rangle ,\langle n_{2}\rangle ,\langle n_{3}\rangle ] ) , \end{aligned}$$

25b $$\begin{aligned} \rho _{2}(\omega )= & {} - \frac{1}{ \pi } Im ( G^{r}_{2,11}[\omega , \langle n_{1}\rangle ,\langle n_{2}\rangle ,\langle n_{3}\rangle ]), \end{aligned}$$

25c $$\begin{aligned} \rho _{3}(\omega )= & {} - \frac{1}{ \pi } Im ( G^{r}_{3,11} [\omega , \langle n_{1}\rangle ,\langle n_{2}\rangle ,\langle n_{3}\rangle ]). \end{aligned}$$

Then, the total DOS of the triple quantum-dot is given by the addition of LDOS of each QD:

26 $$\begin{aligned} \rho (\omega ) = \rho _{1}(\omega ) + \rho _{2}(\omega ) + \rho _{3}(\omega ). \end{aligned}$$

## Results

We now discuss the transport properties within the Andreev regime. Within this regime, the range for the Fermi energy and QD levels is restricted in the range of the superconductor gap, $\Delta$. We denoted *r* as the ratio of leads coupling $\Gamma _S/\Gamma _N$ and assumed that the quantum-dot levels are spin degenerate, $\epsilon _{d i, \sigma } = \epsilon _{d i}$ (for $i = 1,2,3$). In addition, we have introduced the parameter $\eta$, which describes the separation of the levels $\epsilon _{d 1}$ and $\epsilon _{d 3}$ from the level $\epsilon _{d 2} = \epsilon _d$. We have supposed that the energy levels of the side dots (QD1 and QD3) are located symmetrically with respect to the energy level of the central dot (QD2), i.e. $\epsilon _{d 1} = \epsilon _{d} + \eta$ and $\epsilon _{d 3} = \epsilon _{d} - \eta$. Furthermore, unless stated otherwise, we set the coupling between the two side dots to the central dot to be symmetric ( $t_{12} = t_{32} = t$ ), and we consider two regimes: interferometric regime (when *t* is very small in respect to $\Gamma$ ) and the molecular regime (when *t* is very near to the value of $\Gamma$ ). In the linear response regime, the chemical potentials of the leads are set to zero, $\mu _N = \mu _S = 0$. On the other side, in the non-linear regime we set the chemical potential of the leads as $\mu _N = e V$ and $\mu _S = 0$, therefore, $\mu _N-\mu _S=eV$, where *V* is the applied voltage. In addition, we assume in all our calculations that $k_B \tau = 0$, where $\tau$ is the temperature. Finally, we assume that the intradot Coulomb interaction is equal at all quantum dots, i.e., $U_m = U$, for $m=1,2,3$. In what follows, we rename $\Gamma _N=\Gamma$.

### Noninteracting case

Let us first consider the limit where the Coulomb interaction is neglected, $U_i=0$ for $i = 1, 2, 3$.

**Figure 2:**
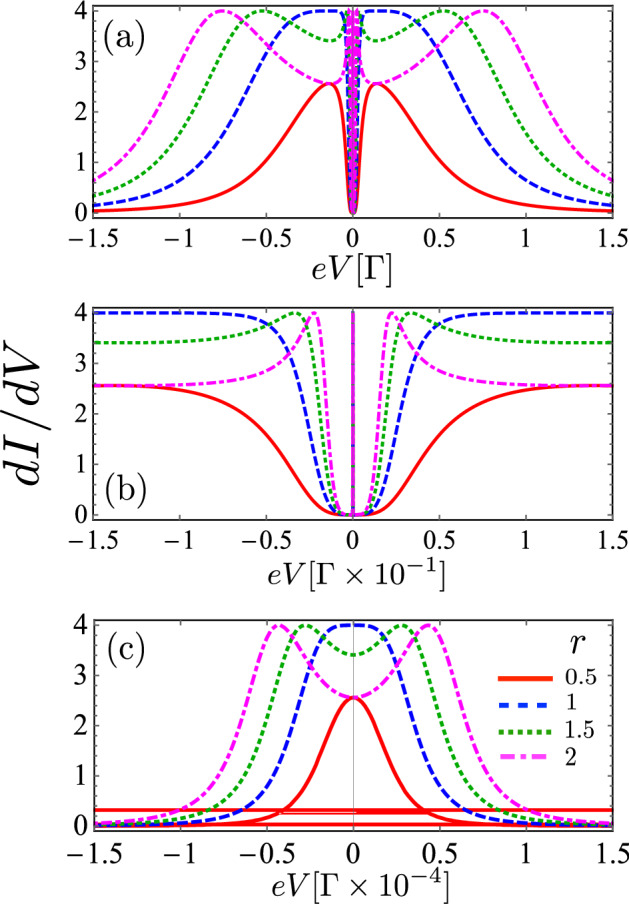
Differential conductance in the noninteracting case, calculated as a function of the bias voltage when $\epsilon _d=0$ and indicated values of *r*: $r= 0.5 (red)$, $r= 1.0 (blue)$, $r= 1.5(green)$, $r= 2.0 (magenta)$. Fixed parameters: $\eta = 0.001 \Gamma$, $t = 0.1 \Gamma$. (**b**) Closeup of central peak presented in panel (**a**). (**c**) Closeup of central peak presented in panel (**b**).

**Figure 3:**
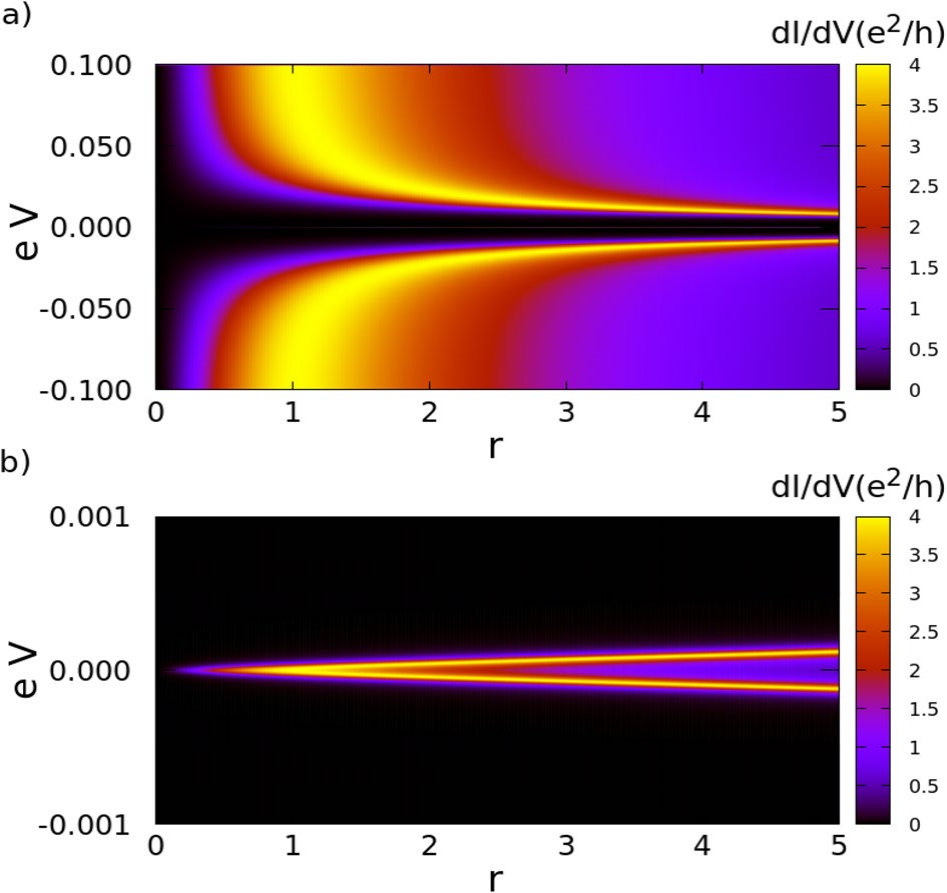
Density plot of differential conductance vs *r* and *eV* in the noninteracting case, when $\epsilon _d = 0 \Gamma$ in the applied bias range between (**a**) $-0.1 \Gamma$ and $0.1 \Gamma$ (**b**) Closeup of the central peak in the figure (**a**), in the applied bias range between $-0.001 \Gamma$ and $0.001 \Gamma$.

In Fig. 2 we show the differential conductance as a function of the bias voltage for different values of $r=\Gamma _S/\Gamma _N$, by choosing a small *t* (interferometric regime) and an even smaller value of $\eta$ in order to investigate the Dicke resonances. We can observe that when *r* does not exceed a certain critical value, the differential conductance exhibits one very narrow central peak and two broader and smaller side peaks symmetrically located with respect to the zero energy. However, for higher values of *r*, we observe that *dI*/*dV* presents six peaks in this interval of energy. In other words, these peaks split as *r* increases from a specific critical value (see inset in Fig. 2). Also, we note from Fig. 3 that the spacing of these peaks increases with the value of *r* when *eV* is very near zero and conversely, they move closer when the range of *eV* increases. In order to have a better understanding of Fig. 2, we will analyze the effect of *r* and $\eta$ on the differential conductance. For that purpose we will study the expression for $T_A$ given by Eq. (23), in the limit of $\Delta \rightarrow \infty$ with $\tilde{\omega }= \omega - \epsilon _d$, for which we obtain,

27 $$\begin{aligned} T_A = \frac{r^2 \Gamma ^4 (\tilde{\omega }^2 - \eta ^2)^2 }{S} \end{aligned}$$

with

$$\begin{aligned} S= 4 \Gamma ^2 \tilde{\omega }^2 \left( \tilde{\omega }^2 - \eta ^2 - 2 t^2 \right) ^2 + \frac{\left( 4 \tilde{\omega }^2 \left( \tilde{\omega }^2 - \eta ^2 - 2 t^2 \right) ^2 - \left( 1 + r^2 \right) \Gamma ^2 \left( \tilde{\omega }^2 - \eta ^2 \right) ^2 \right) ^2 }{ 4 (\tilde{\omega }^2 - \eta ^2)^2}. \end{aligned}$$

From Eq. (27) we can deduce that the roots of $T_A$ are $\tilde{\omega } = \pm \eta$. In the case of symmetric coupling with the leads ($\Gamma _S = \Gamma$), the Andreev transmittance is given by the expression:

28 $$\begin{aligned} T_A = \frac{ \Gamma ^4 (\tilde{\omega }^2 - \eta ^2)^4}{ \Gamma ^4 \left[ \tilde{\omega }^2 - \eta ^2 \right] ^4 + 4 \tilde{\omega }^4 \left[ (\tilde{\omega }^2 - \eta ^2 - 2 t^2 \right] ^4 }. \end{aligned}$$

Equation (28) shows that in the transmittance function there are resonant peaks appearing at $\tilde{\omega } = \pm \sqrt{\eta ^2 + 2 t^2}$, $\tilde{\omega } = 0$, and two Fano antiresonances located at $\tilde{\omega } = \pm \eta$. The narrow central peak in the transmittance may be considered as a long-lived (subradiant) state, while the other two peaks correspond to short-lived (superradiant) states. Since the central peak is located at zero, the width of the central peak is defined by the value of $\eta$. For small values of $\eta$, the width of the central peak becomes much narrower. With $\eta$ increasing, the width of the central line also increases, while the two satellite peaks become broader with $\eta$. This effect resembles the Dicke effect in atomic physics, where a strong narrow emission line appears when the distance between atoms is smaller than the Fermi wavelength of the corresponding radiation. In the present case, the difference of energy between the levels, $\eta$, plays the role of the distance between atoms.

On the other hand, when we consider an asymmetric coupling to the leads, for instance, $r = 2$, the Andreev transmittance function is given by Eq. (29) :

29 $$\begin{aligned} T_A = \frac{\alpha ^4 \Gamma ^4}{ \left[ \tilde{\omega } -\frac{\sqrt{3 \Gamma }}{2} \alpha \right] ^2 \left[ \tilde{\omega }+ \frac{\sqrt{3 \Gamma }}{2} \alpha \right] ^2 + \alpha ^4 \Gamma ^4}, \end{aligned}$$

where $$\alpha = \frac{(\tilde{\omega }^2 - \eta ^2)}{(\tilde{\omega }^2 - \eta ^2 - 2 t^2 )}$$

This function presents two Fano antiresonances when $\alpha = 0$, that is to say at $\tilde{\omega } = \pm \eta$, and six peaks are symmetrically located on either side of the zero-energy at $\omega = \pm \frac{\sqrt{3}}{2} \alpha$ and $\omega = \pm \frac{\sqrt{3}}{2} \alpha$. To gain a more clear physical insight into the dependence of $T_A$ on $\eta$, we analyze the limit $\eta = 0$. The Hamiltonian $H_{TQD}$ can be diagonalized leading to three effective levels $\omega _1 = \epsilon _d + \sqrt{ 2 t^2}$ , and $\omega _2 = \epsilon _d$ and $\omega _3 = - \sqrt{ 2 t^2}$ . Since the system of three one-level QDs has three molecular-like states (denoted by index 1, 2, and 3 for increasing energy), one could also expect three peaks in the conductance. However, the matrix elements of the coupling between the molecular state $|2\rangle$ and the left and right leads vanish, that is, the molecular state $|2\rangle$ decouples from the leads when $\eta = 0$ and the central peak disappears. The Andreev transmittance shows only two peaks at the positions $\tilde{\omega } = \pm \sqrt{ 2 t^2}$ and it is zero at $\tilde{\omega } = 0$. On the other side, when $\eta \ne 0$, the molecular-like levels $\omega _1$ and $\omega _3$ locate symmetrically on both sides of $\mu _R$ and the AR conductance reveals a well-defined central peak due to two-level Andreev reflection, where the conventional resonant tunneling is forbidden due to $\omega _i (i =1,3)$ in the gap. On other hand, when $\omega _i ( i = 1 , 3 )$ aligns with the chemical potential of the superconducting lead, $\mu _R=0$, i.e., $\omega _i (i =1,3) = 0$ two side-peaks appear in ${\tilde{\omega }} = \pm \sqrt{\eta ^2 + 2 t^2}$ due to the Andreev reflection (AR) through a single level. When this happens, an electron coming from the left lead with the energy $\epsilon _d$ can tunnel into the i-state of the QD, leaving a hole propagating back to the i-state in the QD and the creation of a Cooper pair in the right superconducting lead.

**Figure 4:**
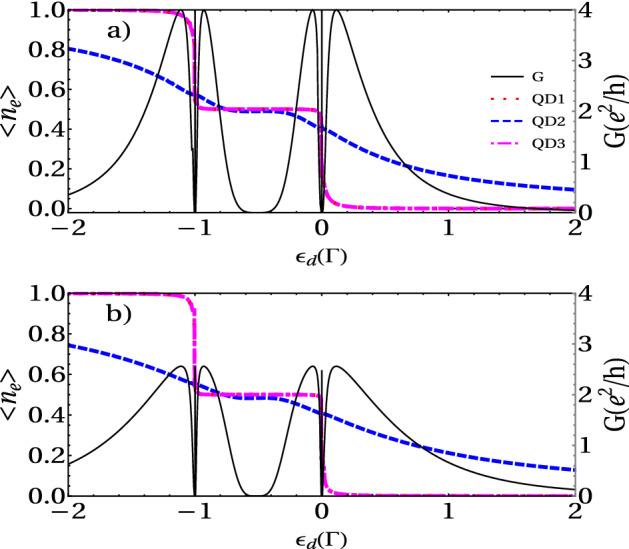
Electronic occupation and linear conductance of a TQD system calculated as a function of the dot’s level energy for (**a**) $r = 1$ and (**b**) r = 2. Fixed parameters: $U = 1 \, \Gamma$, $t = 0.1 \Gamma$, and $\eta = 0.001 \Gamma$.

**Figure 5:**
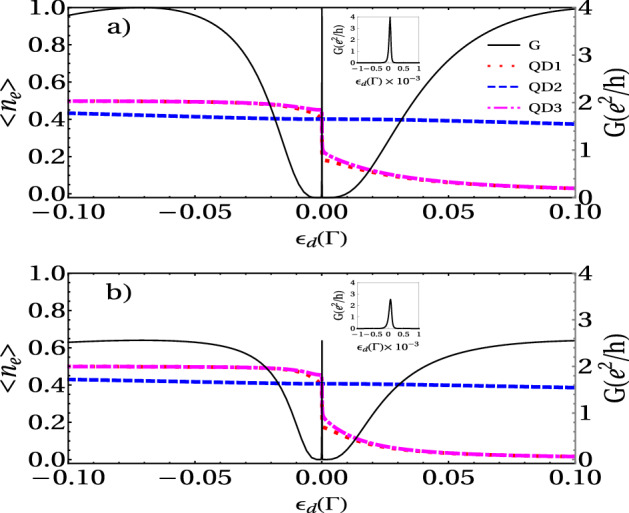
Electronic occupation and linear conductance of a TQD system calculated as a function of the dot’s level energy around $\omega = 0 \Gamma$, for (**a**) $r = 1$ and (**b**) r = 2. Fixed parameters: $U = 1 \, \Gamma$, $t = 0.1 \Gamma$, and $\eta = 0.001 \Gamma$.

**Figure 6:**
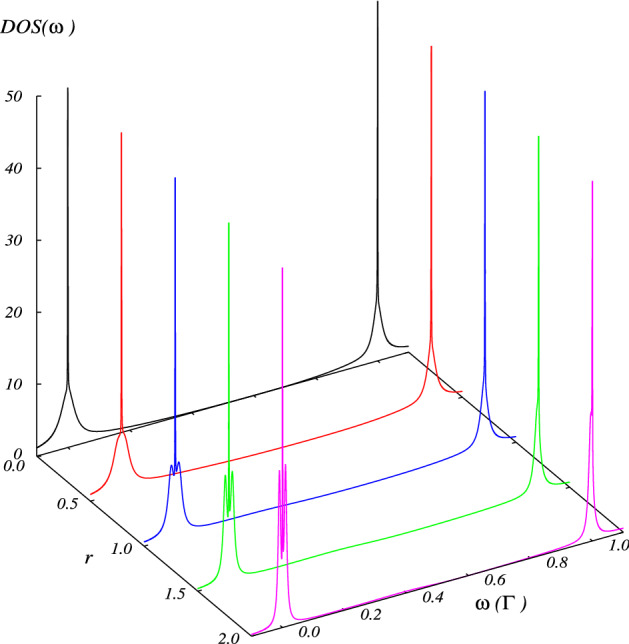
Total DOS in the interacting case, calculated as a function of the energy and *r* for indicated values of rate of coupling: $r= 0$ (black), $r= 0.5$ (red), $r= 1.0$ (blue), $r= 1.5$ (green), $r= 2.0$ (magenta). Fixed parameters: $U = 1 \Gamma$, $\epsilon _d = 0$ , $\eta = 0.001 \Gamma$, $t = 0.1 \Gamma$.

**Figure 7:**
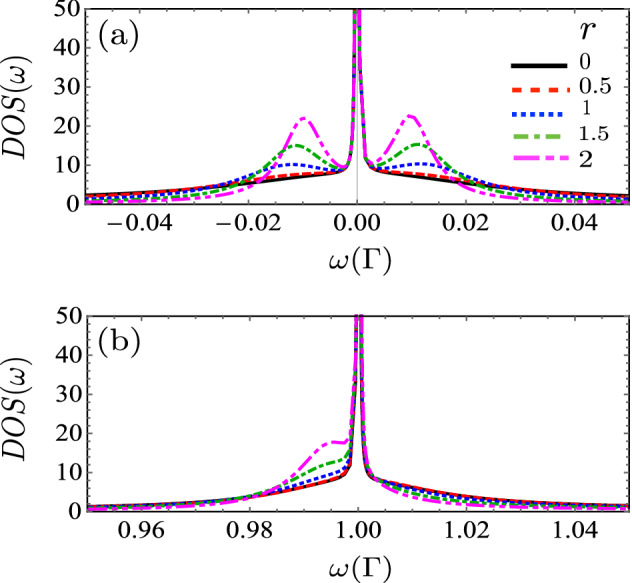
Total DOS calculated as a function of the energy in the interacting case ( $U = 1 \Gamma$), when $\epsilon _d = 0$ is in a range of energy very near (**a**) $\omega = 0 \Gamma$ and (**b**) $\omega = 1 \Gamma$ , and the indicated values of the rate of coupling: $r= 0$ (black), $r= 0.5$ (red), $r= 1.0$ (blue), $r= 1.5$ (green), $r= 2.0$ (magenta). Fixed parameters: $\eta = 0.001 \Gamma$, $t = 0.1 \Gamma$.

### Interacting case

#### Andreev conductance vs gate voltage

In this section, we study the impact of the electronic charging induced by intradot Coulomb interaction on the transport properties of the TQD system in the linear regime. Figures 4 and 5 display the occupation and linear conductance versus the QD energy level $\epsilon _d$ for the interferometric regime ( *t*, $\eta \ll \Gamma$). We can observe that the Coulomb interaction splits the Dicke spectrum into two sets symmetric with respect to the electron-hole symmetry point $\epsilon _d = U/2$, and their centers are located approximately at $\epsilon _d = 0$ and $-U$. The graph of DOS in Fig. 5 confirms this behavior. Besides, four Fano antiresonances in the conductance appear at the electron-hole symmetry due to the destructive quantum interference. Additionally, we can observe the occupation numbers’ features to determine the corresponding linear conductance behavior. It is remarkable that the occupation number presents a staircase-like form, with abrupt changes around $\epsilon _d = 0$ and $\epsilon _d = -U$. This behavior of the charge is due to the Dicke-like spectrum, as we can see in the DOS (Fig. 6), in which a structure of levels with super-tunneling (broad states) and sub-tunneling states (sharp states) develops around $\omega =0$ and $\omega =U$. As $\varepsilon _d$ or $\varepsilon _d+U$ fall slightly below the Fermi energy, the sharp sub-tunneling state completely enters the Fermi sea, and consequently, the charge changes abruptly.

The DOS in Fig. 7 may be written roughly as:

30 $$\begin{aligned} \rho (\omega )\approx \frac{1}{\pi } \sum _{\alpha }\big (\frac{\Gamma _{+}}{(\omega -e_ {\alpha })^2+\Gamma _{+}^2}+\frac{\Gamma _{-}}{(\omega -e_{\alpha })^2+\Gamma _{-}^2}\big ). \end{aligned}$$

In the limit when $\eta \rightarrow 0$, the second term in the sum tends to a Dirac-$\delta$ function. Then, by integrating the above equation at Fermi energy and zero temperature, we obtain:

31 $$\begin{aligned} n_{di} (\varepsilon _d)\approx \frac{1}{2}\theta (\mu -\varepsilon _d)+\frac{1}{2}\theta (\mu -\varepsilon _d-U). \end{aligned}$$

From Eq. (31), we can understand the charge behavior as a function of the energy level $\varepsilon _d$. As $\varepsilon _d$ decreases and falls below the Fermi energy $\mu$, the charge jumps abruptly in steps of 1/2. Besides, since each of these steps in the occupation graph reveals electronic tunneling, three more peaks in the linear conductance plot appear. Each step in electron occupation represents an electron filling from the left normal lead, which occurs when $\epsilon _{di}$ or $\epsilon _{di} + U$ lines up with $\mu _N = \mu _S$. From Eq. (8) one can understand that the intra-level interaction results in an energy level splitting, in the simpler case with only one central QD ($t=0$) : from the original one single-electron spin-degenerate level $\epsilon _d$ splitting into two spin-degenerate levels, $\epsilon _d$ with the probability $1 - \langle n \rangle$ and $(\epsilon _d + U )$ with the probability $\langle n \rangle$. Of particular interest are the sharp peaks seen at both $\epsilon _d = 0$ and $\epsilon _d = -U$. At $\epsilon _d = 0$, $\epsilon _d$ lines up with the Fermi surface $\mu$ (here we have set $\mu _N = \mu _S = \mu = 0$), so $\langle n \rangle$ jumps from 0 to 0.5, describing the first electron filling; then each of both levels, $\epsilon _d$ and $\epsilon _d + U$ , has $50 \%$ probability of being occupied. At $\epsilon _d = -\Gamma$, $\epsilon _d + U$ lines up with the Fermi surface $\mu$, and $\langle n \rangle$ jumps from 0.5 to 1, describing the second electron filling; then level $\epsilon _d + U$ has $100\%$ probability of being occupied while level $\epsilon _d$ has $0\%$ probability.

Additionally, Fig. 5 displays a zoom of the Andreev conductance vs the energy level for $r = 1$ and $r = 2$ around $\epsilon _d = 0$. We can see that as *r* increases, the height of the peaks decreases and, on the contrary, their width increases, in the same way as in the non-interacting case. Besides, as we can see in this figure, for small values of $\eta$, the central peak’s width becomes sharper. Moreover, the insets in the above figure show the details of the sharp resonances. This structure of resonances resembles the Dicke resonance in the optical emission spectra of atoms.

#### Differential Andreev conductance vs bias voltage

**Figure 8:**
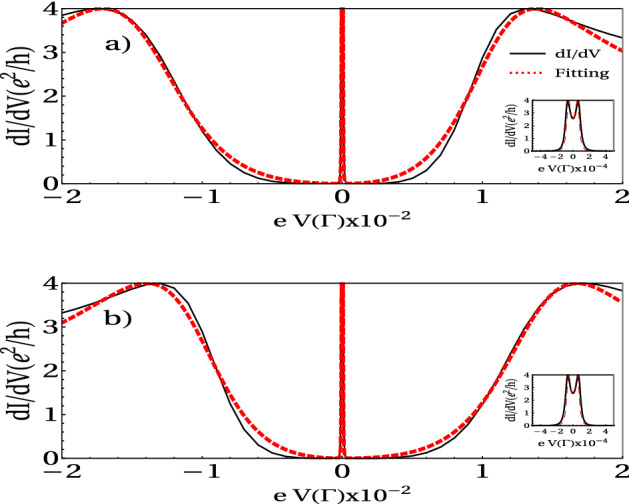
(**a**) The differential conductance (black line) as a function of the bias voltage of a TQD system and the fitting (red line) by the sum of the Fano and BW functions when $\epsilon _d = 0.000075 \Gamma$ $r=2$. (**b**) Differential conductance (solid line) calculated as a function of the bias voltage of a TQD system and the proposed fitting (dashed line) when $\epsilon _d = -1.000075 \Gamma$ and $r = 2$. The zoom of both figures shows the differential conductance and the fitting proposed by the function BW in the low energy limit. Fixed parameters: $U = 1 \, \Gamma$, $t = 0.1 \Gamma$, $\eta = 0.001 \Gamma$.

Next, we investigate the effect of the electronic charging induced by intra-dot Coulomb interaction on the AR process within the non-equilibrium regime. Figure 8 displays the differential conductance as a function of the bias voltage. The central peak, appearing near $eV=0$, is split due to the proximity effect to the superconductor (Andreev reflection). When $r=2$ in this figure, one again observes the splitting of the central peak, but now the separation of each of these peaks from the $e V = 0$ is no longer symmetrical as in the non-interacting case. In addition, if we choose $\epsilon _d= 0$ the height of the peaks decreases as *r* increases, similarly to the non-interacting case (c.f. Fig. 2b), but in Fig. 8 we chose to plot *dI*/*dV* the value of $\epsilon _d$ in which the differential conductance is maximum, so that effect is not observable.

The shape of the differential conductance may be understood by noticing the quantum interference among the electron trajectories entering and leaving the side-attached quantum dots. These interferences give rise to the so-called Fano-Andreev antiresonances^5–7^. On the other hand, the states of two side-attached quantum dots interfere with each other, giving rise to a Dicke-like effect.

**Figure 9:**
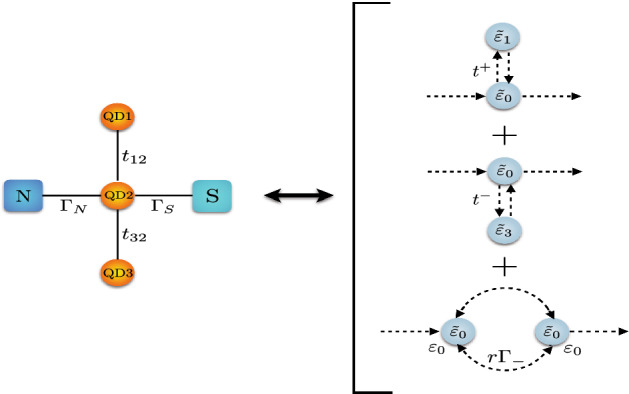
Scheme of the Andreev processes: equivalent path for the Fano-Andreev and Dicke-Andreev transmission. The complete process can be divided in two Fano effects and a transmission through equivalent quantum-dots in series.

The equation for *dI*/*dV* may be written as a superposition of two Fano and Breit–Wigner like line-shapes:

32 $$\begin{aligned} \frac{dI}{dV}\approx \frac{1}{1+q_{+}^2}\frac{(\xi _{+}+q_{+})^2}{\xi _{+}^2+1}+\frac{1}{1+q_{-}^2}\frac{(\xi _{-}+q_{-})^2}{\xi _{-}^2+1}+\frac{4r^2}{|\varepsilon ^2-r^2|^2}, \end{aligned}$$

where $\xi _{\pm }=(V\pm q_{\pm }\Gamma _{S{_\pm }})/\Gamma _{S{_\pm }}$ , $\varepsilon =(V+i\Gamma _{-})/\Gamma _{-}$, and $\Gamma _{-}=\eta ^2/\Gamma _N$. It is worth noting that the last term in the above equation does not contain adjustable parameters. The above equation is explained in Fig. 9, where the first terms take into account the destructive interference given by the Fano-Andreev effect. The last term represents a process equivalent to the transmission through a double quantum-dot in a series configuration with a coupling given by $r\eta$. The fitting of the above equation is shown in Fig. 8 (red line). Similar behavior of the differential conductance can be found in the a quantum dot coupled to a topological superconductor nanowire. In this case, tuning the non-local gate produce the hybridization of the two topological states in the superconductor producing a split of the zero bias peak in the differential conductance^38^.

## Summary

In summary, we have investigated the electronic transport through a triple quantum-dot device coupled to normal and superconductor leads. The main focus was to study the Andreev features appearing in both the differential conductance and QD occupations under Coulomb correlations in the QDs. Within the interferometric regime (small *t*), we have studied the impact on the sub-gap transport properties by varying the coupling ratio with the leads, *r*, when the level spacing between two side-QDs and the central QD is small $\eta \ll 1$. We found that the conductance, both in the interacting and in the non-interacting case, shows the presence Fano-Andreev reflactions and a sharp central peak resembling the Dicke resonance in the optical emission spectra of atoms, in which the role of the distance between atoms is played by the space in energy levels $\eta$. In particular, in the interacting case we show a splitting due to the Andreev reflection of the subradiant and superradiant quasiparticles states whenever the coupling ratio exceeds the certain critical value. On other hand, we explain the shape of the differential conductance near of zero-bias voltage as a result of the two equivalent quantum interference processes: the first, among the electron trajectories entering and leaving the side-attached quantum dots, the one that giving rise, to the so-called Fano-Andreev antiresonances, and the second, the interference between the states of two side-attached quantum dots, the one that giving rise to a Dicke-like effect.

Additionally, we found that when we consider the intradot interaction, the Dicke spectrum split into two symmetric sets equidistant to the electron-hole symmetry point. On the other hand, dramatic changes in the charge are produced when a subradiant state falls below the Fermi energy. This property could be used to store charge in quantum dots.

## Appendix

### $T_A$ in the limit $\Delta \gg \epsilon$

Here we present the Andreev transmittance for a TQD between normal and superconducting leads in the limit $\Delta \gg \epsilon$ and zero inter-dot tunneling ($t = 0$).

33 $$\begin{aligned} T(\omega )= & {} 2 T_A(\omega ) + T_Q(\omega ) \end{aligned}$$

34 $$\begin{aligned} T_A(\omega )= & {} \Gamma _N^2 ( |G_{ 12}^{r} |^2 ) \end{aligned}$$

35 $$\begin{aligned} T_Q= & {} \Gamma _{N} \Gamma _{S} \, \bar{\rho } \left( |G_{ 11}^{r}|^2 + |G_{ 12}^r|^2 - \frac{2 \Delta }{|\epsilon |} Re \left[ G_{ 11}^r ( G_{ 12}^{r} )^ *\right] \right) \end{aligned}$$

with

36 $$\begin{aligned} \bar{\rho }(\omega ) = \frac{| \omega | \theta (| \omega | - \Delta )}{\sqrt{ \omega ^2 - \Delta ^2}}. \end{aligned}$$

On other side

37 $$\begin{aligned} G_{12}(\omega ) = \frac{i \Gamma _S \Delta \rho (\omega )}{2 \omega D}, \end{aligned}$$

where

$$\begin{aligned} \rho (\omega ) = \frac{| \omega | \theta (| \omega | - \Delta )}{\sqrt{ \omega ^2 - \Delta ^2}} -i \frac{\omega \theta (\Delta - | \omega | )}{\sqrt{ \Delta ^2 - \omega ^2 }} \end{aligned}$$

and

$$\begin{aligned} D = \left( g_{ 11}^{-1} - \Sigma _{NS} \right) \left( g_{ 22}^{-1} - \Sigma _{NS} \right) + \frac{\Gamma ^2 \Delta ^2 \rho ^2}{4 \omega ^2}, \end{aligned}$$

where $\Sigma _{NS} = -\frac{1}{2} i \left( \Gamma _N + \Gamma _S \rho \right)$ is the self-energy of the leads, and

38 $$\begin{aligned} g_{11}^{-1}= & {} \omega - \epsilon _d - \frac{t^2}{\omega - \epsilon _1}- \frac{t^2}{\omega - \epsilon _3} \end{aligned}$$

39 $$\begin{aligned} g_{22}^{-1}= & {} \omega - \epsilon _d - \frac{t^2}{\omega + \epsilon _1}- \frac{t^2}{\omega + \epsilon _3} \end{aligned}$$

with $$\epsilon _1 = \epsilon _d + \eta$$ and $$\epsilon _3 = \epsilon _d - \eta$$

If the real part of $\rho (\omega )$ is named “a” and his imaginary part “b”, we can write the above equation as:

40 $$\begin{aligned} G^{r}_{12}(\omega ) = \frac{ (a - b \,i) \Gamma _S \Delta /2 \omega }{\left[ c + d \,i \right] }, \end{aligned}$$

where

41 $$\begin{aligned} \begin{aligned} c&= \frac{\Gamma _S^2 \Delta ^2}{4 \omega ^2}(a^2 - b^2) - \frac{1}{4} \left( \Gamma _N + \Gamma _S a \right) ^2 \\&+ \left( g_{ 11} - b \, \Gamma _S /2 \right) \left( g_{ 22}^{-1} - b \, \Gamma _S /2\right) \\ d&= \frac{ \left( \Gamma _N + a \, \Gamma _S \right) }{2} \left( g_{ 11}^{-1} + g_{ 22}^{-1} - b \Gamma _S \right) \\&+ \frac{\Gamma _S^2 \Delta ^2 a \,b}{2 \omega ^2 }. \end{aligned} \end{aligned}$$

Consequently, we have

42 $$\begin{aligned} |G_{12} |^2= \frac{\Gamma _S \Delta }{2 \omega } \frac{ (a^2 + b^2 ) }{\left[ c^2 + d^2 \right] }. \end{aligned}$$

#### Symmetric Coupling $\Gamma _N = \Gamma _S$

Substituting these expressions in the equation to $G_{12}$ and considering $t \ne 0$, and $\Gamma _N = \Gamma _S = \Gamma$ at the limit of $\Delta \gg \omega$ we have:

43 $$\begin{aligned} T_A= & {} \Gamma _{N}^2|G_{12}^{\sigma } |^2\\= & {} \Gamma _{N}^2|G_{12}^{\sigma } |^2= \frac{ \Gamma ^4 (\omega ^2 - \eta ^2)^4}{ \Gamma ^4 \left[ \omega ^2 - \eta ^2 \right] ^4 + 4 \omega ^4 \left[ (\omega ^2 - \eta ^2 - 2 t^2 \right] ^4 }. \nonumber \end{aligned}$$

Then, the first maximum occurs when $\omega = 0$ and the two other maxima values of $T(\epsilon )$ occur when $\omega = \pm \sqrt{\eta ^2 + 2 t^2}$.

#### Asymmetric Coupling $\Gamma _S=2\Gamma$

Substituting these expressions in the equation to $G_{12}$, considering $t \ne 0$, and $\Gamma _S = 2 \Gamma$ at the limit of $\Delta \gg \omega$ we have:

44 $$\begin{aligned} T_A= & {} \Gamma _{N}^2|G_{12}^{\sigma } |^2 \\= & {} \frac{ \Gamma ^4}{ \left[ \frac{\omega \Gamma \left( \omega ^2 - \eta ^2 -2 t^2 \right) }{ (\omega ^2 - \eta ^2)} \right] ^2 + \left[ \omega ^2 \frac{ (\omega ^2 - \eta ^2 - 2 t^2 )^2}{(\omega ^2 - \eta ^2)^2} -\frac{5 \Gamma ^2}{4} \right] ^2 }. \nonumber \end{aligned}$$

In this case because of this functional structure, $T_A$ presents six peaks located symmetrically each side of $\omega =0$. As before when the coupling to the leads was symmetric, the zeros of $T(\omega )$ occur when $\omega = \pm \eta$. At the particular case $t = 0$ and $\eta = 0$, we have:

45 $$\begin{aligned} T_A(\omega ) = \frac{ \Gamma ^4 }{ \left[ \Gamma ^2 \epsilon ^2 + (\omega ^2 - 5\Gamma ^2/4)^2 \right] }. \end{aligned}$$

Then, the maximum value of $T(\omega )$ occurs close to $\omega = \pm \Gamma$:

$$\begin{aligned} T_A( \pm \Gamma )= & {} \frac{ \Gamma ^4 }{ \left[ \Gamma ^2 \Gamma ^2 + (\Gamma ^2 - 5\Gamma ^2/4)^2 \right] } \\= & {} \frac{ \Gamma ^4 }{ \left[ \Gamma ^4 + (\Gamma ^2/4)^2 \right] } \\= & {} \frac{ 16 \Gamma ^4 }{ 17 \Gamma ^4 }\\\approx & {} 1. \end{aligned}$$

### Linear and differential conductance in the limit of low temperature

Linear conductance *G* in the sub-gap limit $( e V \ll \Delta )$ is written as:

46 $$\begin{aligned} G = \frac{4 e^2}{h}\int \frac{\partial f}{\partial \omega } T_A(\omega ) d\omega . \end{aligned}$$

If $k_B \tau \rightarrow 0$ then $\frac{\partial f}{\partial \omega } \rightarrow \delta (\omega - \mu )$, so that

47 $$\begin{aligned} G = \frac{4 e^2}{h}\int \delta (\omega - \mu ) T_A(\omega ) d\omega = \frac{4 e^2}{h} T_A(\mu ). \end{aligned}$$

Consequently, the peaks of $T_A$ as a function of the energy $\epsilon$ for a fixed value of $\epsilon _d$ are the same for *G* as a function of $\mu$ for a fixed value of $\epsilon _d$. For this reason, as expected, the graph of *G* in function of the Fermi energy $\mu$, in the case of a quantum dot asymmetric coupling with the leads, presents two peaks as the case of $T_A$ as a function of the energy.

### *dI*/*dV* vs $T_A$

The general form for the electronic current in the sub-gap limit $( e V \ll \Delta )$ is

48 $$\begin{aligned} I = \frac{2 e}{h} \int d\omega \, T_A \left[ f_L(\omega - e V) - f_L(\omega + e V)\right] . \end{aligned}$$

In the limit of low temperature $k_B \tau \rightarrow 0$

49 $$\begin{aligned} I= & {} \frac{2 e}{h} \int \ d\omega \, T_A \left[ \Theta ( e V - \omega ) - \Theta (-\omega - e V)\right] \end{aligned}$$

50 $$\begin{aligned} I= & {} \frac{2 e}{h} \int d\omega \, T_A \left[ 1- \Theta ( \omega - e V) - 1 + \Theta (\omega + e V)\right] \end{aligned}$$

51 $$\begin{aligned} I= & {} \frac{2 e}{h} \int d\omega \, T_A \left[ \Theta ( \omega + e V) - \Theta (\omega - e V)\right] \end{aligned}$$

52 $$\begin{aligned} I= & {} \frac{2 e}{h} \int ^{e V}_{-e V} d\omega \, T_A ( \omega ). \end{aligned}$$

Then

53 $$\begin{aligned} \frac{d I}{d V}= & {} \frac{2 e}{h} \int d\omega \, T_A \frac{\partial }{\partial V}\left[ \Theta ( \omega + e V) - \Theta (\omega - e V)\right] \end{aligned}$$

54 $$\begin{aligned} \frac{d I}{d V}= & {} \frac{2 e}{h} \int d\omega \, T_A \left[ \delta ( \omega + e V) + \delta (\omega - e V)\right] \end{aligned}$$

55 $$\begin{aligned} \frac{d I}{d V}= & {} \frac{2 e^2}{h} \left[ T_A (- e V) + T_A( e V) \right] = \frac{4 e^2}{h} T_A( e V). \end{aligned}$$

Consequently, for a fixed value of $\epsilon _d$, the peaks of $T_A$ as a function of *eV* are the same as $\partial I /\partial V$ in function of *eV*.

## References

[1] Guevara ML, Claro F, Orellana PA (2003). Ghost Fano resonance in a double quantum dot molecule attached to leads. Phys. Rev. B.

[2] Lu H, Lu R, Zhu BF (2006). Tunable Fano effect in parallel-coupled double quantum dot system. J. Phys. Condens. Matter.

[3] Baranski J, Domanski T (2012). Decoherence effect on the Fano lineshapes in double quantum dots coupled between normal and superconducting leads. Phys. Rev. B.

[4] Ding GH, Kim CK, Nahm K (2005). Fano resonance in electron transport through parallel double quantum dots in the Kondo regime. Phys. Rev. B.

[5] Calle M, Pacheco M, Martins GB, Apel VM, Lara GA, Orellana PA (2017). Fano-Andreev effect in a T-shape double quantum dot in the Kondo regime. J. Phys. Condens. Matter.

[6] Calle AM, Pacheco M, Orellana PA (2013). Fano effect and Andreev bound states in T-shape double quantum dots. Phys. Lett. A.

[7] Barański J, Zienkiewicz T, Barańska M, Kapcia KJ (2020). Anomalous Fano resonance in double quantum dot system coupled to superconductor. Sci. Rep..

[8] Gómez I, Domínguez-Adame F, Orellana PA (2004). Fano-like resonances in three-quantum-dot Aharonov–Bohm rings. J. Phys. Condens. Matter.

[9] Ye C-Z, Li Z-J, Nie Y-H, Liang J-Q (2008). Rashba spin-orbit interaction induced spin-polarized Andreev-reflection current through a double Aharonov-Bohm interferometer. J. Appl. Phys..

[10] Trocha P, Barnaś J (2007). Quantum interference and Coulomb correlation effects in spin-polarized transport through two coupled quantum dots. J. Phys. Rev. B.

[11] Sztenkiel D, Świrkowicz R (2007). Interference effects in a double quantum dot system with inter-dot Coulomb correlations. J. Phys. Condens. Matter.

[12] Liu Y-S, Chen H, Yang X-F (2007). Transport properties of an Aharonov–Bohm ring with strong interdot coulomb interaction. J. Phys. Condens. Matter.

[13] Dicke RH (1953). The effect of collisions upon the Doppler width of spectral lines. Phys. Rev..

[14] Dicke RH (1954). Coherence in spontaneous radiation processes. Phys. Rev..

[15] Brandes T (2005). Coherent and collective quantum optical effects in mesoscopic systems. Phys. Rep..

[16] Shahbazyan TV, Raikh ME (1994). Two-channel resonant tunneling. Phys. Rev. B.

[17] Shahbazyan TV, Ulloa SE (1998). Localized states in a strong magnetic field: Resonant scattering and the Dicke effect. Phys. Rev. B.

[18] Vorrath T, Brandes T (2003). Dicke effect in the tunnel current through two double quantum dots. Phys. Rev. B.

[19] Wunsch B, Chudnovskiy A (2003). Quasistates and their relation to the Dicke effect in a mesoscopic ring coupled to a reservoir. Phys. Rev. B.

[20] Orellana PA, Ladrón de Guevara ML, Claro F (2004). Controlling Fano and Dicke effects via a magnetic flux in a two-site Anderson model. Phys. Rev. B.

[21] Trocha P, Barnaś J (2008). Dicke-like effect in spin-polarized transport through coupled quantum dots. J. Phys. Condens. Matter.

[22] Trocha P, Barnaś J (2008). Kondo–Dicke resonances in electronic transport through triple quantum dots. Phys. Rev. B.

[23] Vernek E, Orellana PA, Ulloa SE (2010). Suppression of Kondo screening by the Dicke effect in multiple quantum dots. Phys. Rev. B.

[24] Wang Q, Xie H, Nie Y-H, Ren W (2013). Enhancement of thermoelectric efficiency in triple quantum dots by the Dicke effect. Phys. Rev. B.

[25] Sun Q-F, Wang J, Lin T-H (1999). Resonant Andreev reflection in a normal-metal-quantum-dot-superconductor system. Phys. Rev. B.

[26] Yu Z, Qing-Feng S, Tsung-Han L (2001). Effect of intra-dot coulomb interaction on Andreev reflection in normal-metal/quantum-dot/superconductor system. Commun. Theor. Phys. (Beijing, China).

[27] Siqueira EC, Cabrera GG (2010). Andreev tunneling through a double quantum-dot system coupled to a ferromagnet and a superconductor: Effects of mean-field electronic correlations. Phys. Rev. B.

[28] Trocha P, Barnaś J (2014). Spin-polarized Andreev transport influenced by Coulomb repulsion through a two-quantum-dot system. Phys. Rev. B.

[29] Trocha P, Barnaś J (2017). Spin-dependent thermoelectric phenomena in a quantum dot attached to ferromagnetic and superconducting electrodes. Phys. Rev. B.

[30] Bai L, Zhang R, Duan C-L (2010). Andreev reflection tunneling through a triangular triple quantum dot system. Phys. B.

[31] Bardeen J, Cooper LN, Schrieffer JR (1957). Microscopic theory of superconductivity. Phys. Rev..

[32] Orellana PA, Lara GA, Anda EV (2006). Kondo and Dicke effect in quantum dots side coupled to a quantum wire. Phys. Rev. B.

[33] de Guevara ML, Orellana PA (2006). Electronic transport through a parallel-coupled triple quantum dot molecule: Fano resonances and bound states in the continuum. Phys. Rev. B.

[34] Bai L, Wu Y-J, Wang B (2010). Andreev reflection in a triple quantum dot system coupled with a normal-metal and a superconductor. Phys. Status Solidi B.

[35] Xu W-P, Zhang Y-Y, Wang Q, Li Z-J, Nie Y-H (2016). Thermoelectric effects in triple quantum dots coupled to a normal and a superconducting leads. Phys. Lett. A.

[36] Glodzik S, Wójcik KP, Weymann I, Domański T (2017). Interplay between electron pairing and Dicke effect in triple quantum dots structures. Phys. Rev. B.

[37] Hubbard J, Flowers BH (1963). Electron correlations in narrow energy bands. Proc. R. Soc. Lond. A.

[38] Zhang H, Liu DE, Wimmer M, Kouwenhoven LP (2019). Next steps of quantum transport in Majorana nanowire devices. Nat. Commun..

